# Plant Growth-Promoting Bacteria Influence Microbial Community Composition and Metabolic Function to Enhance the Efficiency of *Hybrid pennisetum* Remediation in Cadmium-Contaminated Soil

**DOI:** 10.3390/microorganisms12050870

**Published:** 2024-04-26

**Authors:** Zhao-Jin Chen, Meng-Lu Li, Shan-Shan Gao, Yu-Bo Sun, Hui Han, Bai-Lian Li, Yu-Ying Li

**Affiliations:** Overseas Expertise Introduction Center for Discipline Innovation of Watershed Ecological Security in the Water Source Area of the Mid-line Project of South-to-North Water Diversion, College of Water Resource and Environment Engineering, Nanyang Normal University, Nanyang 473061, China; zhaojin_chen@163.com (Z.-J.C.);

**Keywords:** cadmium (Cd), phytoremediation, bacterial community and function, high-throughput sequencing, non-targeted metabolomic

## Abstract

The green and efficient remediation of soil cadmium (Cd) is an urgent task, and plant-microbial joint remediation has become a research hotspot due to its advantages. High-throughput sequencing and metabolomics have technical advantages in analyzing the microbiological mechanism of plant growth-promoting bacteria in improving phytoremediation of soil heavy metal pollution. In this experiment, a pot trial was conducted to investigate the effects of inoculating the plant growth-promoting bacterium *Enterobacter* sp. VY on the growth and Cd remediation efficiency of the energy plant *Hybrid pennisetum*. The test strain VY-1 was analyzed using high-throughput sequencing and metabolomics to assess its effects on microbial community composition and metabolic function. The results demonstrated that *Enterobacter* sp. VY-1 effectively mitigated Cd stress on *Hybrid pennisetum*, resulting in increased plant biomass, Cd accumulation, and translocation factor, thereby enhancing phytoremediation efficiency. Analysis of soil physical-chemical properties revealed that strain VY-1 could increase soil total nitrogen, total phosphorus, available phosphorus, and available potassium content. Principal coordinate analysis (PCoA) indicated that strain VY-1 significantly influenced bacterial community composition, with Proteobacteria, Firmicutes, Chloroflexi, among others, being the main differential taxa. Redundancy analysis (RDA) revealed that available phosphorus, available potassium, and pH were the primary factors affecting bacterial communities. Partial Least Squares Discriminant Analysis (PLS-DA) demonstrated that strain VY-1 modulated the metabolite profile of *Hybrid pennisetum* rhizosphere soil, with 27 differential metabolites showing significant differences, including 19 up-regulated and eight down-regulated expressions. These differentially expressed metabolites were primarily involved in metabolism and environmental information processing, encompassing pathways such as glutamine and glutamate metabolism, α-linolenic acid metabolism, pyrimidine metabolism, and purine metabolism. This study utilized 16S rRNA high-throughput sequencing and metabolomics technology to investigate the impact of the plant growth-promoting bacterium *Enterobacter* sp. VY-1 on the growth and Cd enrichment of *Hybrid pennisetum*, providing insights into the regulatory role of plant growth-promoting bacteria in microbial community structure and metabolic function, thereby improving the microbiological mechanisms of phytoremediation.

## 1. Introduction

With the rapid development of China’s industry, soil heavy metal pollution has become increasingly severe and widespread, with cadmium (Cd) being the most severely polluted among all elements [1,2]. Cd is highly ecotoxic and can accumulate in various plants, posing a serious threat to crop productivity and safety. It is considered a significant pollutant in agricultural soil [3].

In the field of heavy metal-contaminated soil remediation, phytoremediation has emerged as a green and sustainable method because it does not harm the soil ecological environment and avoids secondary pollution [4,5]. *Hybrid pennisetum* has become an excellent cellulosic energy plant due to its rapid growth and strong stress resistance. It exhibits a robust ability to absorb various heavy metals and is considered one of the most promising energy plants in China, having been extensively researched for the remediation of heavy metal-contaminated soil [6,7,8]. Choosing *Hybrid pennisetum* as a remediation plant not only allows for the remediation of heavy metal-contaminated farmland and abandoned mining areas but also enables the stems of the revitalized plant to be used for fermentation to produce fuel ethanol. This approach realizes both soil remediation and subsequent resource processing [9]. However, remediation plants like *Hybrid pennisetum* often exhibit slow growth, reduced biomass, and low heavy metal transfer rates under heavy metal stress conditions, severely limiting phytoremediation efficiency.

Plant growth-promoting bacteria (PGPB), which inhabit the rhizosphere soil, can mitigate the toxicity of heavy metal ions to plants by leveraging their own resistance systems [10]. They can also alter the form and mobility of heavy metals, directly or indirectly promoting plant growth and enhancing plant resistance to heavy metals. Consequently, PGPB play a crucial role in strengthening the process of phytoremediation of soil heavy metal pollution [11]. Plant-microbial joint remediation has garnered significant attention in the field of heavy metal-contaminated soil remediation as it leverages the complementary strengths of plants and microorganisms [10,12].

Soil microorganisms are integral to the soil environment, participating in complex biological and ecological processes and exerting a significant influence on plant growth and phytoremediation efficiency [13]. The effectiveness of plant-microbe joint remediation largely hinges on the adaptability of inoculated PGPB to environmental changes and their impact on the composition and metabolic functions of indigenous microbial communities. In recent years, omics technologies (such as high-throughput sequencing and metabolomics) have been employed in plant-microbial joint remediation, providing valuable insights into the mechanisms underlying PGPB-mediated improvements in phytoremediation [14,15,16]. Therefore, this study utilized 16S rRNA high-throughput sequencing and metabolomics technology to investigate the effects of the plant growth-promoting bacterium *Enterobacter* sp. VY-1 on the microbiological mechanism of phytoremediation enhancement from the perspectives of diversity and metabolic function.

## 2. Materials and Methods

### 2.1. Test Materials

The test strain was selected from plant growth-promoting bacteria preserved in the laboratory, identified as belonging to the genus *Enterobacter*, and named *Enterobacter* sp. VY-1. *Enterobacter* sp. VY-1 exhibits plant growth-promoting properties such as phosphorus solubilization, potassium solubilization, siderophore production, and IAA production, along with good tolerance to Cd. For detailed information, refer to Liu et al. [17]. *Hybrid pennisetum* seeds were procured from Shuyang County Tuojing Horticulture Co., Ltd. (Suqian, China). The potting soil was obtained from non-polluted soil in the pomegranate garden of Nanyang Normal University. The basic physical and chemical properties of the soil can be found in Liu et al. [17]. The soil was air-dried naturally after removing impurities such as leaves and stones, and then passed through a 20-mesh sieve for later use. CdSO_4_·8H_2_O was added in proportion to achieve a Cd ion content of 20 mg·kg^−1^ in the soil, thoroughly mixed, and allowed to equilibrate for 30 days.

### 2.2. Pot Experiment

The soil was filled into plastic flowerpots, with each pot containing 0.75 kg of soil. *Hybrid pennisetum* seeds of uniform size were sown in the plastic flowerpots. For greenhouse cultivation, the potted plants were randomly placed to ensure consistent growing conditions. Thinning was initiated when the *Hybrid pennisetum* seedlings reached a height of 5 cm, leaving three seedlings in each pot. *Enterobacter* sp. VY-1 was inoculated into liquid LB medium, cultured to the logarithmic growth phase, centrifuged to collect the cells, and then resuspended in sterile deionized water to achieve a cell concentration of 1 × 10^8^ cfu·mL^−1^. The test strains were inoculated into the pots on the 30th, 45th, and 60th days after *Hybrid pennisetum* germination, with an inoculation volume of 10 mL per strain, while the control group was inoculated with an equal amount of sterile water. After 90 days, the *Hybrid pennisetum* plants were harvested, and rhizosphere soil samples were collected.

### 2.3. Determination of Cd Content and Analysis of Soil Physical-Chemical Properties

The collected plants were dried at 80 °C until a constant weight was achieved, and the dry weight was measured. The sample was ground with a mortar, and 0.1 g (±0.0002 g) of the plant sample was accurately weighed into a polytetrafluoroethylene crucible. The hydrochloric acid-nitric acid-hydrofluoric acid-perchloric acid method was used for microwave digestion, followed by filtration, and the Cd content was determined by inductively coupled plasma optical emission spectrometer (ICP-OES). Additionally, 5.0 g (±0.05) of soil sample was accurately weighed into a 50 mL centrifuge tube, and DTPA (pH = 7.30 ± 0.05) extract was added. After shaking and centrifugation, the supernatant was filtered with a 0.22 μm filter, and ICP-OES was used to measure the content of DTPA-Cd. Soil pH was measured using a potentiometric method at a soil-to-water ratio of 1:5. The available phosphorus content in soil samples was determined using the 0.5 mol L^−1^ NaHCO_3_ leaching-molybdenum antimony spectrophotometry method, while flame photometry was employed to determine the available potassium content. The hydrolyzed nitrogen content in soil samples was determined using the alkaline hydrolysis diffusion method.

### 2.4. High-Throughput Sequencing

A 0.5 g sample of rhizosphere soil was accurately weighed, and total DNA was extracted using the FastDNA SPIN Kit soil DNA extraction kit (MP Biochemicals, Solon, OH, USA). The DNA concentration and purity were determined using a micro-UV spectrophotometer. PCR amplification of the V3~V4 conserved region of the bacterial 16S rRNA gene was performed using universal primers 338F and 338R [18]. Sequence determination was carried out using the Illumina MiSeq PE300 high-throughput sequencer (Illumina, San Diego, CA, USA), and the PE reads obtained by Illumina sequencing were spliced based on the overlap relationship. Sequence quality control and filtering were implemented, followed by OTU cluster analysis and species taxonomy analysis. Diversity index calculation and sequencing depth detection were performed using Mothur 1.30.2, Qiime 1.9.1, etc., based on OTU. Statistical analysis of community structure at various classification levels was conducted based on taxonomic information.

### 2.5. Metabolomic Analysis

A 1 g soil sample was accurately weighed, 20 μL of internal standard and 1 mL of 50% methanol solution were added, and the sample was ground, vortexed, and centrifuged to collect the supernatant. It was then filtered using a 0.22 μm organic phase pinhole filter, and stored at −80 °C for subsequent analysis. Non-targeted metabolome analysis was conducted using an Agilent 7890B Infinity gas chromatograph (Agilent Co., Santa Clara, CA, USA) coupled with an Agilent 5977A GC-MS. The measurement conditions followed those described by Zhang et al. [19]. Principal component analysis (PCA) was utilized to observe the clustering patterns of metabolites in all samples and the repeatability of samples within the group. Pairwise orthogonal partial least squares-discriminant analysis (OPLS-DA) was employed to enhance the discrimination between groups and screen metabolic markers. Metabolites with VIP values > 1 and *p* < 0.05 were identified as differential metabolites between groups. The databases METLIN (https://metlin.scripps.edu/,accessed on 20 April 2023) and HMDB (http://www.hmdb.ca/, accessed on 20 April 2023) were consulted to identify these differential metabolites.

### 2.6. Data Analysis

Data analysis of soil physical-chemical properties, heavy metal content, high-throughput sequencing, and other data was performed using SPSS 17.0. Differences between groups were assessed using *t*-tests and one-way analysis of variance.

## 3. Results

### 3.1. Effects of Tested Strains on Growth and Cd Accumulation of Hybrid pennisetum

As shown in Table 1, compared to the uninoculated control, inoculation with *Enterobacter* sp. VY-1 increased the dry weight of the shoots and roots by 25.23% and 32.91%, respectively, indicating that *Enterobacter* sp. VY-1 effectively alleviates Cd-induced stress on the growth of *Hybrid pennisetum*. The Cd content in the shoots and roots correlated with the dry weight results. Inoculation with *Enterobacter* VY-1 led to a 35.48% and 24.25% increase in the Cd content in the shoots and roots, respectively. Based on the dry weight and Cd content in different tissues, *Enterobacter* sp. VY-1 increased the Cd accumulation in the shoots and roots by 64.35% and 51.66%, respectively, thereby enhancing phytoremediation efficiency. The transfer factor (TF) represents the ratio of the heavy metal content in the above-ground parts of the plant to that in the underground part. It reflects the plant’s ability to transfer heavy metals from roots to above-ground parts after absorption. The transfer factor increased from 0.15 to 0.16 after inoculation with *Enterobacter* sp. VY-1, a 6.67% increase.

### 3.2. Effects of Tested Strains on Soil Physicalchemical Properties

Plant growth-promoting bacteria can enhance the content of mineral nutrients in the soil, thus improving the absorption of these nutrients by crops. Inoculation with *Enterobacter* sp. VY-1 increased soil total nitrogen, total phosphorus, available phosphorus, and available potassium contents by 3.95%, 4.33%, 19.23%, and 2.31%, respectively (Table 2).

### 3.3. Effect of Tested Strains on Bacterial Community Composition

**Figure 1:**
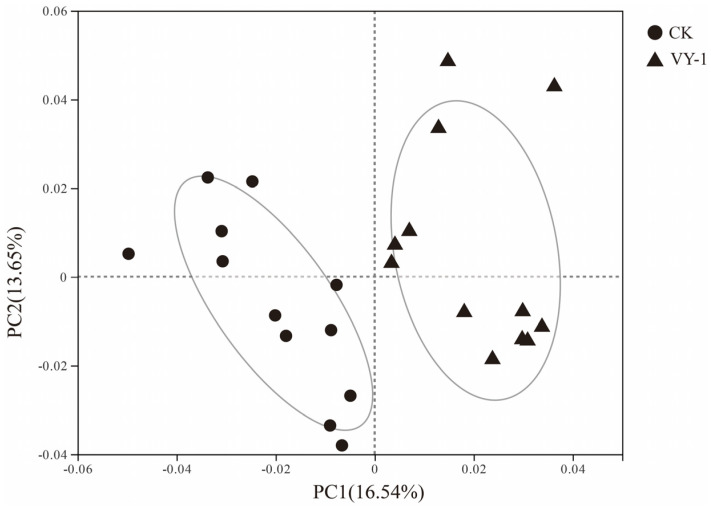
PCoA results of bacterial community diversity.

The high-throughput sequencing results revealed 14,951 OTUs in the two sample groups, with 6461 OTUs shared between CK and VY-1, 1218 OTUs unique to CK, and 811 OTUs unique to VY-1. To investigate the impact of *Enterobacter* sp. VY-1 on the bacterial community structure diversity of *Hybrid pennisetum* rhizosphere soil under Cd stress, principal coordinate analysis (PCoA) was employed to assess the dissimilarities between the two sample sets. As depicted in Figure 1, PC1 contributed to 16.54% of the variance, while PC2 contributed to 13.65%. The CK sample clustered on the left side of the PCoA plot, whereas the *Enterobacter* sp. VY-1 inoculated sample clustered on the right side, indicating that the bacterial treatment influenced the bacterial community composition. Subsequent ANOSIM analysis (Analysis of Similarities) revealed a significant intergroup difference with a *p*-value of 0.003.

**Figure 2:**
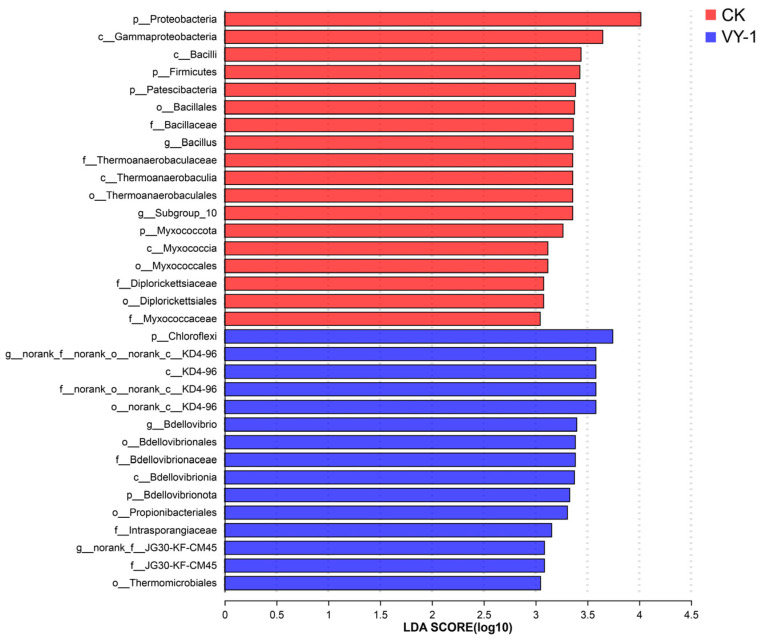
LefSe analysis identifying the most differentially abundant taxa.

LefSe (Linear discriminant analysis Effect Size) software (version 1.0) was utilized to examine variations in rhizosphere soil bacterial community composition across different treatment levels. As illustrated in Figure 2, 33 taxa exhibited significant differences in relative abundance with an LDA score ≥ 3. At the phylum level, significant differences were observed in the CK treatment group for Proteobacteria, Myxococcota, Patescibacteria, and Firmicutes, while Chloroflexi and Bdellovibrionota exhibited significant differences following inoculation with *Enterobacter* sp. VY-1. At the order level, there were significant differences among the CK treatment groups in Diplorickettsiales, Thermoanaerobaculales, Myxococcales, and Bacillales. In the treatment with *Enterobacter* sp. VY-1, significant differences were noted in Thermomicrobiales, KD4-96, Propionibacteriales, and Bdellovibrionales. At the genus level, significant differences were observed among the CK treatment groups for *Bacillus* and *Subgroup-10*, while *Bdellovibrio*, *KD4-96*, *JG30*-*KF-CM45* exhibited significant differences among the *Enterobacter* sp. VY-1 treatment groups.

**Figure 3:**
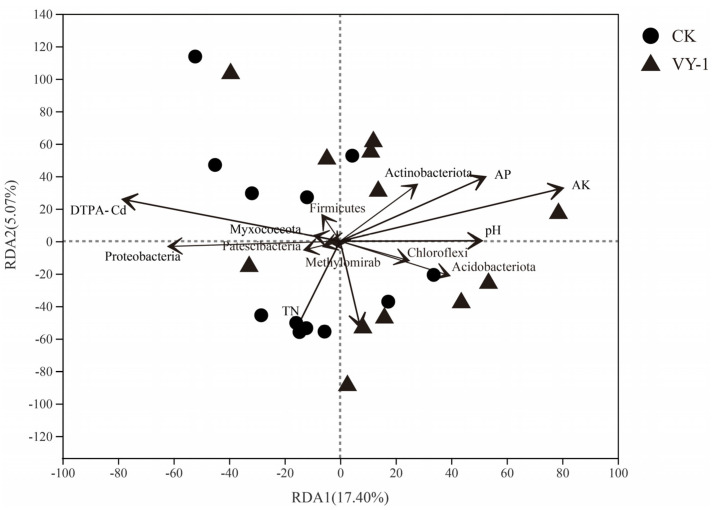
RDA ordination biplot between bacteria communities and environmental factors.

Redundancy analysis (RDA) was employed to assess the relationship between bacterial flora and environmental factors. RDA1 explained 17.40%, and RDA2 explained 5.07% of the variance (Figure 3). Utilizing the envfit function to test the significance of environmental factors, it was determined that AK (r^2^ = 0.342, *p* = 0.013) and DTPA-Cd (r^2^ = 0.254, *p* = 0.044) significantly influenced the composition of the rhizosphere soil bacterial community of *Hybrid pennisetum* (*p* < 0.05). The order of influence of soil physical and chemical factors on microbial community composition is AK > DTPA-Cd > AP > pH > TN > TP.

### 3.4. Effect of Test Strains on Metabolic Function

**Figure 4:**
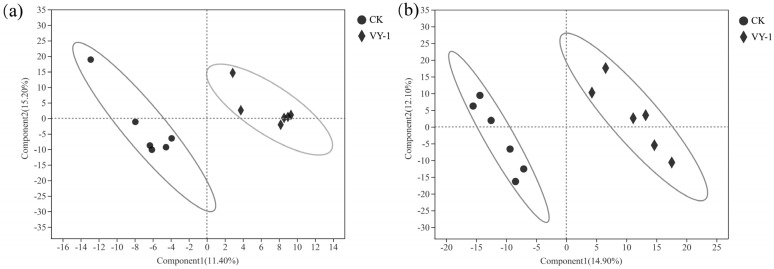
PLS-DA analysis chart of cationic (**a**) and anionic (**b**) metabolites in the rhizosphere soil of *Hybrid pennisetum*.

To explore the alterations in soil metabolites in the rhizosphere of *Hybrid pennisetum* under Cd stress induced by inoculation with the plant growth-promoting bacterium VY-1, Partial Least Squares Discriminant Analysis (PLS-DA) was conducted. In the PLS-DA diagram, the abscissa and ordinate represent the explanatory power of the first (Component1) and second (Component2) principal components, respectively. The PLS-DA results reveal that the metabolites in positive and negative ion modes of both the CK and *Enterobacter* sp. VY-1-inoculated groups are distinctly segregated on opposite sides of the PLS-DA diagram, indicating a significant impact of the inoculation treatment on the metabolite profile of *Hybrid pennisetum* rhizosphere soil (Figure 4). The verification results of the PLS-DA model demonstrate that R^2^ is greater than Q^2^ in both positive and negative ion modes, with the intercepts of the Q^2^ regression line and the Y vertical axis being −0.2420 and −0.2205, respectively. This indicates a good fit of the model, high predictability, and suitability for subsequent data analysis.

**Figure 5:**
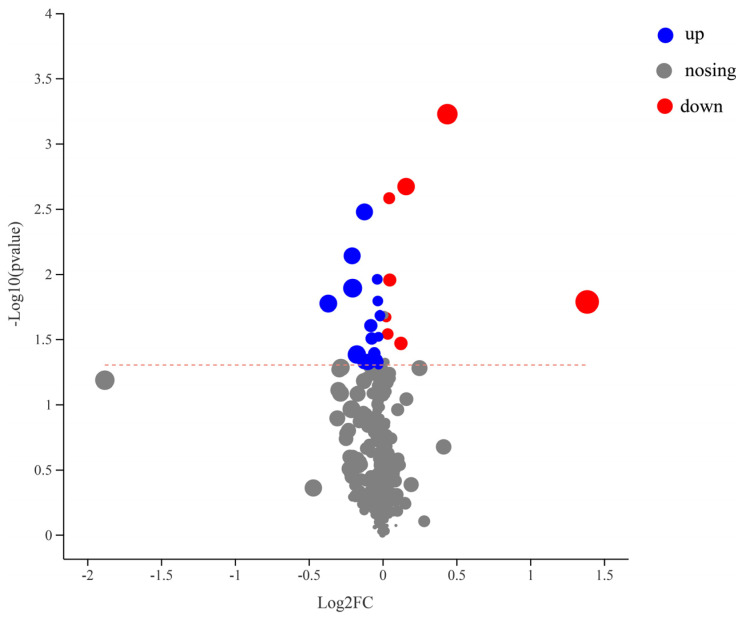
Volcano plot showing differential metabolites between groups.

The variable weight value (variable important in projection, VIP) extracted from the OPLS-DA analysis was utilized, with VIP > 1 considered significant. Additionally, a fold change (FC) value greater than 1 or less than 1 was used as a screening criterion. The T test standard of *p* < 0.05 was employed to identify differential metabolites. The results are depicted in Figure 5. In comparison with the control group, a total of 27 differential metabolites were identified in the *Enterobacter* sp. VY-1 treatment group. Among these, metabolites exhibiting up-regulated expression differences mainly included Poly-g-D-glutamate, Methyl 9,10-epoxy-12,15-octadecadienoate, and 1-Formylneogrifolin, among others, totaling 19 metabolites. Conversely, metabolites with down-regulated expression differences mainly comprised Petasitenine, 3-Hydroxy-10′-apo-b,y-carotenal, (3beta, 5alpha, 6alpha, 22E, 24R)-Ergosta-7,9(11),22-triene-3,5,6-triol, and 8 others. These findings indicate that inoculation with *Enterobacter* sp. VY-1 regulated the metabolites in Pennisetum rhizosphere soil to varying degrees under Cd stress.

Analysis of the metabolic functional pathways of the differentially expressed metabolites using KEGG revealed that inoculation with *Enterobacter* sp. VY-1 primarily affected primary metabolic pathways, particularly those related to Metabolism and Environmental Information Processing. Among these, the metabolic pathway was the most prominently impacted. The secondary classification of KEGG metabolic pathways included membrane transport, lipid metabolism, amino acid metabolism, metabolism of terpenoids and polyketides, and nucleotide metabolism.

**Figure 6:**
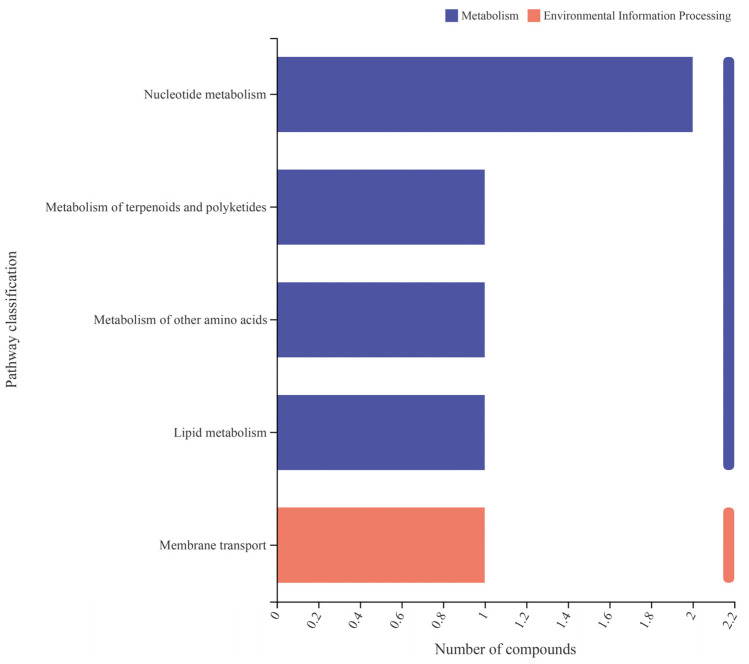
Analysis of KEGG metabolic functional pathways of differentially expressed metabolites.

Fisher’s exact test was employed to conduct enrichment analysis of the 27 significantly different metabolites. The Benjamini and Hochberg (BH) method was utilized for multiple correction of the *p* values, with a threshold of 0.05 for the corrected *p* value. KEGG pathways meeting this criterion were considered significantly enriched in the metabolic set. The results revealed that the 27 differential metabolites were mainly enriched in 6 metabolic pathways, including glutamine and glutamate metabolism, alpha-linolenic acid metabolism, pyrimidine metabolism, purine metabolism, zeatin biosynthesis, and ABC transporters (Figure 6). Notably, four pathways were significantly enriched (*p* < 0.05), namely glutamine and glutamate metabolism, alpha-linolenic acid metabolism, pyrimidine metabolism, and purine metabolism. Glutamine and glutamate metabolism exhibited the highest enrichment factor among these pathways.

## 4. Discussion

As a high-biomass energy plant, *Hybrid pennisetum* has been extensively studied for its ability to remediate heavy metal-contaminated soil. Hou et al. [20] conducted a comparative analysis of the potential of four herbaceous energy plants, namely switchgrass, barley, arundodis, and *Hybrid pennisetum*, for remediating soil heavy metals. They found that *Hybrid pennisetum* exhibited the greatest remediation potential. However, under the stress of heavy metals, the biomass of *Hybrid pennisetum* decreases, slowing down its growth and reducing its efficiency in remediation [6,8]. Plant growth-promoting bacteria are commonly employed in various phytoremediation systems to aid in enhancing phytoremediation [9,11]. Wu et al. [7] isolated the strain *Bacillus megaterium* BM18-2 from *Hybrid pennisetum* plants. The plant growth-promoting characteristics of this strain, including its ability to produce IAA and dissolve phosphorus, among other growth-promoting abilities, resulted in an increase in the biomass of *Hybrid pennisetum* by 83.1% and Cd accumulation by 28.6%. This indicates that plant growth-promoting bacteria have a positive effect on remediating heavy metal pollution in *Hybrid pennisetum*. Similarly, in this experiment, *Enterobacter* sp. VY-1, which exhibits strong growth-promoting ability, was inoculated into *Hybrid pennisetum*. The pot experiment demonstrated that the inoculation treatment increased plant biomass and Cd accumulation by 25.23% to 32.91%, respectively, and 64.35% to 51.66%, effectively alleviating Cd-induced stress and improving phytoremediation efficiency. Moreover, the transfer factor (TF) increased from 0.15 to 0.16, indicating that *Enterobacter* sp. VY-1 enhanced the ability of *Hybrid pennisetum* to transfer Cd absorbed from the roots to the shoots [21]. Heavy metals can significantly interfere with the utilization efficiency of plant nutrients such as N, P, and S, thereby limiting the growth of remediation plants. Plant growth-promoting bacteria activate the content of effective mineral elements in the soil, which is an important mechanism for enhancing plant growth and phytoremediation efficiency [9,11]. The test strains isolated in this experiment all possess the ability to produce siderophores, dissolve phosphorus, and dissolve potassium. Pot experiments revealed that these strains increased the total nitrogen, total phosphorus, available phosphorus, and available potassium content in the rhizosphere soil of *Hybrid pennisetum* by 3.95%, 4.33%, 19.23%, and 2.31%, respectively. This finding aligns with the study by Kamal et al. [6], which concluded that the plant growth-promoting bacterium *Bacillus megaterium* BM18-2 primarily acts as a biofertilizer to enhance the Cd remediation efficiency of *Hybrid pennisetum*.

Soil microbial communities constitute an integral part of the soil ecosystem, exerting a significant influence on plant growth, development, and the efficiency of Cd remediation by plants [22,23]. In the process of plant-microbial joint remediation, inoculation with plant growth-promoting bacteria can impact not only plant growth directly but also the soil microbial community, thereby influencing the function of the rhizosphere microbiome [24,25]. In this experiment, PLS-DA analysis of *Hybrid pennisetum* rhizosphere soil samples revealed significant changes in the bacterial community following inoculation with the VY-1 strain. This finding is consistent with the findings of Kong et al. [24], suggesting that plant growth-promoting bacteria can directly or indirectly impact the soil microbial community structure. Analysis of differential bacteria showed that at the phylum level, the dominant phyla were Proteobacteria, Myxococcus, Patellae, Firmicutes, and Chloroflexi. Bourceret et al. [26] also demonstrated that Proteobacteria, Acidobacteria, and Bacteroidetes are the predominant bacterial phyla in heavy metal-contaminated soil. Liu et al. [27] observed that inoculation of rhizosphere bacteria, such as *Rhodococcus erythropolis*-NSX2, not only enhances the growth of *Sedum sedum* and its Cd accumulation but also leads to an increase in the numbers of indigenous heavy metal-tolerant bacteria like actinomycetes and Bradyrhizobia. Following inoculation with the VY-1 strain, the relative abundance of Actinobacteria, Acidobacteria, Chloroflexi, and Bacteroidetes increased. Numerous studies have highlighted the growth-promoting properties associated with most members of these bacterial phyla [28,29,30]. For instance, Bacteroidetes, an important indicator for assessing soil ecosystem health, exhibits potential biological control functions [31]. Many genera of Actinobacteria possess plant growth-promoting abilities and heavy metal resistance, thereby promoting plant growth and enhancing plant resistance to peroxidative damage under metal stress [28,32]. Moreover, the majority of Acidobacteria phylum members play crucial roles in soil organic-matter recycling and ecological environment construction, exhibiting significant growth-promoting effects [33]. Therefore, inoculation with plant growth-promoting bacteria in this study may enhance the relative abundance of beneficial microorganisms in the rhizosphere soil of *Hybrid pennisetum* [21,34]. Analyzing the factors influencing the composition of the bacterial community, it was found that AK, AP, DTPA-Cd, and pH are the physical-chemical factors that predominantly contribute to the community structure. This finding aligns with the study by Zhao et al. [35], indicating that plant growth-promoting bacteria can influence soil bacterial community composition by releasing organic acids and dissolving phosphorus and potassium.

The addition of exogenous microorganisms can influence the metabolic activities of soil microorganisms. Utilizing LC-MS non-targeted metabolomics technology to investigate changes in soil metabolites under heavy metal stress induced by inoculation of plant growth-promoting bacteria is crucial for analyzing the joint remediation mechanism of plants and microorganisms [14,36]. Zuluaga et al. [37] demonstrated that inoculation with *Enterobacteriaceae* leads to specific alterations in the metabolism and function of tomato rhizosphere soil under adverse stress conditions. By enhancing the catabolism of amino acids by rhizosphere microorganisms, it can enhance the tolerance of tomato plants and promote plant growth. Similarly, our study employed PLS-DA analysis to illustrate that inoculation with the functional strain VY-1 can impact metabolites in the rhizosphere soil of *Pennisetum* spp., resulting in the identification of 27 differential metabolites, among which 19 were up-regulated and eight were down-regulated. This indicates that inoculating the VY-1 strain in the rhizosphere of *Hybrid pennisetum* enhances the activity of surrounding microorganisms and induces certain changes in soil metabolites, thereby mitigating the toxic effects of heavy metals on microorganisms and *Hybrid pennisetum* [38]. Cluster analysis of these differential metabolites revealed an increase in lipids, amino acids, carbohydrates, and other metabolites following inoculation with the VY-1 strain. Zhang et al. [39] utilized non-targeted metabolomics technology to analyze the metabolites of *Ganoderma lucidum* under different concentrations of Cd stress and found that *Ganoderma lucidum* can alleviate the toxic effects of Cd by enriching lipid metabolites. Wang et al. [40] demonstrated that amino acids can play a functional role in reducing oxidative damage, regulating cell penetration, and maintaining plant growth. Carbohydrates serve as the primary source of energy for plants, and changes in their content can serve as an important indicator of plant physiological conditions. An increase in carbohydrate content indicates favorable plant growth conditions, and its augmentation significantly enhances the biomass of burdock roots under Cu stress [41]. Through KEGG pathway enrichment analysis of significantly different metabolites, it was observed that metabolic pathways were predominantly affected by inoculation with the VY-1 strain among the primary metabolic pathways. After inoculation with the VY-1 strain, six main metabolic pathways were affected, with four pathways being significantly enriched (*p* < 0.05): glutamine and glutamate metabolism, α-linolenic acid metabolism, pyrimidine metabolism, and purine metabolism. Limami et al. [42] found that aspartate, alanine, and glutamate metabolism can promote plant growth by regulating nitrogen flux and increasing carbon availability.

## 5. Conclusions

Research on the joint remediation mechanism of plant-microorganism systems is crucial for enhancing the practical application of remediating heavy metal-contaminated soil. This study utilized high-throughput sequencing combined with metabolomics omics technology to investigate the microbiological mechanism of *Enterobacter* sp. VY-1, a plant growth-promoting bacterium, in remediating Cd pollution in *Hybrid pennisetum*. The findings revealed that *Enterobacter* sp. VY-1 effectively mitigates Cd-induced stress, increases *Hybrid pennisetum* biomass, Cd accumulation, and transfer factor (TF), thus enhancing phytoremediation efficiency. The primary mechanism involves activating soil mineral nutrients to increase soil total nitrogen, total phosphorus, available phosphorus, and available potassium content. Moreover, it influences the composition of the *Hybrid pennisetum* rhizosphere soil bacterial community, impacting phyla such as Proteobacteria, Myxococcus, Patellae, and Firmicutes. Furthermore, it modulates the metabolite profile of *Hybrid pennisetum* rhizosphere soil, particularly affecting primary metabolic pathways like metabolism and environmental information processing as well as specific pathways such as glutamine and glutamate metabolism, α-linolenic acid metabolism, pyrimidine metabolism, and purine metabolism. Hence, plant growth-promoting bacteria can enhance the efficiency of phytoremediation in heavy metal-contaminated soil by influencing the composition and metabolic functions of microbial communities.

## Figures and Tables

**Table 1:** Effects of inoculation *Enterobacter* sp. VY-1 on biomass, Cd content, accumulation, and TF (translocation factor) in different parts of *Hybrid pennisetum*.

Treatment	Aboveground Biomass (g)	Root Biomass (g)	Aboveground Cd Content (mg·kg^−1^)	Root Cd Content (mg·kg^−1^)	Aboveground Cd Accumulation (μg)	Root Cd Accumulation (μg)	TF
CK	5.39 ± 0.85	0.79 ± 0.15	2.48 ± 0.37	16.74 ± 1.83	13.38 ± 2.77	13.28 ± 2.80	0.15
VY-1	6.75 ± 0.93	1.05 ± 0.08	3.36 ± 1.03	20.80 ± 2.60	21.99 ± 4.08	20.14 ± 1.62	0.16

**Table 2:** Effects of inoculation *Enterobacter* sp. VY-1 on the physicochemical properties of *Hybrid pennisetum* rhizosphere soil.

Treatment	pH	Total Nitrogen (mg·kg^−1^)	Total Phosphorus (mg·kg^−1^)	Available Phosphorus (mg·kg^−1^)	Available Potassium (mg·kg^−1^)
CK	7.09 ± 0.12	1.77 ± 0.10	243.07 ± 9.34	0.026 ± 0.001	108.25 ± 2.5
VY-1	7.13 ± 0.09	1.87 ± 0.19	253.60 ± 19.57	0.031 ± 0.006	110.75 ± 2.7

## Data Availability

The data that support the findings of this study are available on request from the corresponding author.
